# Brand safety: the effects of controversial video content on pre-roll advertising

**DOI:** 10.1016/j.heliyon.2018.e01041

**Published:** 2018-12-19

**Authors:** Steven Bellman, Ziad H.S. Abdelmoety, Jamie Murphy, Shruthi Arismendez, Duane Varan

**Affiliations:** a Ehrenberg-Bass Institute, University of South Australia, Adelaide, Australia; b Department of Business Administration, Faculty of Commerce, Assiut University, Assiut, Egypt; c University of Eastern Finland, Joensuu, Finland; d University of Western Australia, Perth, Australia; e Mediascience, Austin, Texas, USA

**Keywords:** Business, Information science, Psychology, Sociology

## Abstract

Newspapers have reported instances of famous brands' ads running as pre-rolls to terrorist videos on YouTube. Subsequent brand safety fears have led to advertisers pulling their YouTube ads. This study, a lab experiment, tested the effects of program quality and content—particularly violent, sexual or extremist content—on pre-roll ads. The experiment used measures from studies showing significant broadcast TV content effects on mid-roll advertising, using more extreme cable TV content to increase the chances of finding significant effects on pre-roll ads. Overall, the effects were minimal, with no effects on brand attitudes, ad liking, or three ad memory components—encoding, storage, and retrieval. In contrast to research showing program context effects on mid-roll advertising, context effects (e.g., on brand safety) do not seem an issue for pre-roll ads. A brand's reputation might suffer negative effects from pre-roll advertising in other ways, however. A limitation is that this study did not re-test the effects of controversial content on mid-roll advertising.

## 1 Introduction

Industry and academia have long suspected that undesirable ad contexts reduce the positive effects of ads and harm brand reputation. Such “brand safety” issues could affect hundreds of brands and governmental agencies advertising on YouTube channels that promote radical or contentious content such as terrorism, white nationalism, Nazis, pedophilia, conspiracy theories and North Korean propaganda (Murphy et al., 2018). Ads for famous brands, and US government departments (e.g., Department of Transportation and Centers for Disease Control), which ran as pre-rolls to short videos promoting extremist (terrorist) or other controversial content, led to some brands boycotting YouTube ads (Hickman, 2017; Wakabayashi and Maheshwari, 2017). These advertisers had brand safety “guardrails” in place to prevent their ads appearing next to content that contrasts with their brand's values (Murphy et al., 2018). These safety settings failed.

Brand safety failures affect brands in at least three ways. First, if an advertiser becomes aware of this failure, the advertiser often ceases YouTube advertising, at least for a few months. Dropping YouTube advertising prevents the brand from accessing young viewers who are hard to reach with broadcast television. Second, when a brand's pre-roll ad appears in front of controversial content, such as hate speech from a terrorist group, the terrorist group gets income from that advertising. This means the brand gives monetary support to a terrorist group with opposing values. The support may be unintended, but any single instance could have a substantial negative effect on the brand's reputation if it were widely publicized (Nature, 2017). Third, watching the short video content after the ad could negatively affect pre-roll ad effectiveness. This study investigated the third brand safety effect, the effects of extremist and other videos on pre-roll ad effectiveness, measured by brand recall, recognition, ad liking, brand attitude, and purchase intention, as well as by biometrics measures of emotional response.

With over 400 hours of video content uploaded to YouTube every minute, computers have taken over the job of matching videos with sponsoring ads (Maheshwari, 2017). Programmatic buying—computerized media scheduling—saves time and effort when placing spots in programs or on websites (Fulgoni and Lipsman, 2017). The advertiser supplies a list of requirements, such as target audience, visibility, etc., plus the available budget, and the computer hunts for and buys the best spots available that fulfill those requirements. By incorporating behavioral targeting, programmatic buying focuses on delivering the right audience—whenever, wherever and whatever they are watching—rather than using audience proxies such television program demographics.

A programmatic buying danger, however, is that the right audience may see a brand's ads in an unfavorable context. Buyers can control the context of their ads via direct deals, that is, by buying ad space the traditional way, without using computerized programmatic buying. Alternatively, buyers can include brand safety parameters (guardrails) in their programmatic buying list of requirements. This study investigated what these parameters should be. It tested parameters such as rating (e.g., E for extremist) and budget (high vs. low). Since the number of YouTube videos is too large for human classifiers, machine learning could instead assign a rating and identify the likely budget spent on producing an uploaded video (Maheshwari, 2017).

Research of advertising context effects varies. These mixed results could be due to the variables examined, or differences between mid- and pre-roll ads. A mid-roll study found negative effects of broadcast television content on advertising awareness, attitude, and behavior (Bushman, 2005). The behavior measured in that study was coupon choice. As a reward for participating in the experiment, participants could choose up to ten coupons from 40 available for different brands. These coupons were professionally designed and promised one dollar off the purchase price of the brand. Only ten of the 40 brands had been advertised during the experiment, so the dependent variable was the number of coupons chosen for advertised brands. But another study, which tested the effects of extremist YouTube content on pre-roll ads, found no content effects on dimensions of brand attitude (positivity, consideration, honesty, trustworthiness, etc.) (Nature, 2017). This second study, which suggests that even extremist content is not the brand safety danger that many fear, underscores a need for further research.

As in other studies, this study examined brand attitude, as well as ad liking, purchase intention, brand recall and recognition, and biometrics measuring emotional response, to help understand controversial content effects. The results help advertisers make evidence-based programmatic buying decisions and set content parameters, if necessary.

## 2 Theory

The limited capacity for motivated mediated message processing (LC4MP) model (Lang, 2017) helps understand how and why program content might affect video advertising. This theory assumes that, at any moment, people have limited cognitive resources available for media processing. When watching video these available cognitive resources allocate across three memory processes—encoding, storage, and retrieval.

Encoding stores video content attended to into long-term memory (not all content is attended to). How well that encoded content is stored is a function of the second process, storage. Storage describes the forging of links between newly encoded information and old information from memory. Activating this old information from memory requires the third process, retrieval. The success of these three memory processes can be measured by three memory tasks that increase in difficulty (Lang, 2017). *Recognition* is an easy task, as the thing to be remembered is supplied, and the person doing the task just has to answer whether that thing was seen before. Recognition measures encoding. *Cued recall*, which supplies a cue that helps people retrieve an item, is a harder task, and measures storage. The most difficult memory task is free recall, because the person is given no cues to help retrieval. *Free recall* measures retrieval.

When people watch video content, they devote more cognitive resources to encoding than to storage and retrieval. This is because video content is not self-paced like print content but dynamic, continually introducing new information on the screen for potential encoding. Thus, video viewers find it difficult to recall what they have seen, even when supplied with helpful cues (Lang, 2017; Lang et al., 2013).

Even fewer resources are available for storage and retrieval when video content includes novel or relevant stimuli. Biologically relevant stimuli include food and sex, essential for the survival of a species (Lang et al., 2005). Species survival potentially explains why violence is another biologically relevant stimulus (Lull et al., 2018). These kinds of stimuli are automatically attended to and encoded (Öhman et al., 2001), leaving fewer resources than normal for storage and retrieval of other stimuli, such as advertising (Bushman and Bonacci, 2002).

Because of its biological relevance, violence and sex distract encoding resources away from encoding advertising content. Bushman and Bonacci (2002) argue that violence and sex very likely distract resources from storage and retrieval as well. When viewing any type of television program, thinking about the program's plot distracts cognitive resources away from storing and retrieving memories for advertising content. The biological relevance of violent and sexual content potentially increases the likelihood that viewers will think about the program more than they think about the ads. Together, the distracting effect of violent and sexual content on all three memory processes makes it very unlikely that viewers will remember ads seen during programs that feature violence or sex. Potentially, this distraction effect applies to pre-roll ads seen before the program content, as well as to ads seen in mid-roll ad breaks during the content.

Researchers have used program classifications—violent (V), sexual (S), or neutral (G)—to test the effects of controversial broadcast television content on commercials in ad breaks (Bushman and Bonacci, 2002; Bushman, 2005). Bushman and Bonacci (2002) found that viewers not only paid attention to mid-roll ads in G programs but were able to recognize and recall the brands advertised in these ads. When these same commercials were seen in V or S programs, however, brand recognition was lower than it was for the G programs, indicating distraction from encoding. Free brand recall was lower as well, indicating distraction from storage and retrieval. In a later study, Bushman (2005) found negative effects of V, S, or V + S program content on all three memory processes, including storage measured by cued recall. In the same study, he showed that these memory effects mediated the negative effects of controversial content on purchase intention and actual behavior (coupon choice).

The above two studies, however, did not compare memories for ads seen before versus after violent or sexual content. It is unclear from these studies whether the negative effects of violent or sexual content affect pre-roll ads seen before controversial content.

A study that compared memory for content seen before versus during violent news offers evidence for a potential distracting effect of violence on encoding and storage of previously seen content (Lang et al., 1996). When the news content was neutral, content seen before the news and content seen during the news was recognized equally well. But when the news content was violent, the news content seen during the news was recognized significantly better than the content seen before the news. Lang et al.'s (1996) study does not report enough data (e.g., means and standard deviations) to allow us to test whether there was a significant difference between violent versus neutral news content on recognition for content seen before the news. Potentially this difference in recognition, between content seen *before* violent versus neutral news, was insignificant, as in Lang et al.'s study this difference was smaller than the difference for content seen *during* violent versus neutral news.

The authors (Lang et al., 1996) speculated that the violent content may have had a greater effect on storage and retrieval, which generally receive fewer processing resources than encoding when people watch video, but they did not measure cued recall or free recall of the content prior to the news. The present research compares memory for pre-roll ads, all seen before violent or sexual content. The results of this research provide what may be the first test of whether violent or sexual content has a backwards effect on encoding, storage, and retrieval.

### 2.1 Extremist content

The evidence reviewed suggests that violent and sexual content distracts memory for embedded advertising. This distraction is likely to have a forward effect on ads seen after violent or sexual content. But violent or sexual content may also have a backwards interference effect, especially on the storage and retrieval of content, such as previously seen ads. Extremist content, such as from terrorist organizations like ISIS, might also have forward or backwards interference effects on advertising.

To the extent that extremist content is violent (e.g., showing beheadings) then it is likely to have forward and perhaps backward interference effects just like other violent content. But an advertiser could avoid these negative effects of extremist content simply by avoiding advertising spots associated with V-rated content.

Yet extremist content from organizations like ISIS may not show violence. To recruit people to its cause, organizations like ISIS also show positive and inspiring content. Terrorist organization content often contains speeches, including “hate speech,” from leaders or other spokespersons (Murphy et al., 2018; Wakabayashi and Maheshwari, 2017). If such non-violent extremist content has a negative effect, it will most likely be due to generating negative viewer emotions. The content itself could be negative (hate speech) or so opposed to viewer beliefs that it elicits strong negative reactions.

The evaluative space model helps explain how negative emotion could attenuate remembering video advertising (Cacioppo et al., 2012). This theory posits that the body has two emotional response systems, a positive (appetitive) and a negative (aversive) system. Stimuli can activate one or both systems at the same time (Lee and Lang, 2009; Wang and Lang, 2012). If one fears snakes, seeing a snake activates the aversive system. If the aversive system activates more than the appetitive system, the body prepares for withdrawal by increasing arousal in the sympathetic nervous system (SNS) (Cacioppo et al., 2012; Potter et al., 2006). This aroused SNS diverts energy and cognitive resources away from encoding and storage.

Negative stimuli prompt action (withdrawal) more rapidly than positive stimuli because “losses loom larger than gains” (Kahneman and Tversky, 1979). This preparation for action directs processing resources to retrieving vital information about how to escape (Lang, 2017), and storing the negative stimulus for future reference (Lang et al., 2013) before devoting all resources to behavioral action (Cacioppo et al., 2012). Thus, people have strong recall of central elements but little recognition of peripheral elements of fearful scenes, a phenomenon known as “weapon focus” (Christianson, 2017). For these reasons, people should have difficulty remembering peripheral content seen before, during, and after aversive content (Lang et al., 1996, 2013; Wang and Lang, 2012). In summary, extremist content that is perceived as aversive may have a negative effect on memory for pre-roll advertising, even extremist content without violence.

Extremist video content that generates high negative emotional responses will generate high aversive system activation and high SNS arousal, leading to a high behavioral disposition for withdrawal. High levels of SNS arousal are “sticky,” which means they decay slowly over time (Cacioppo et al., 2012; Wang and Lang, 2012). For example, symptoms of SNS arousal after viewing highly arousing content take about four minutes to return to normal (Mattes and Cantor, 1982). Thus, extremist content potentially has a negative effect on advertising seen after the content and may hinder storage and retrieval of content seen prior to extremist content (Lang et al., 1996, 2013).

In Bushman's (2005) investigation, violent and sexual content's memory effects explained its effects on attitude and behavior. But extremist content may affect attitudes and behaviors for a different reason. Even if a negative emotional response to extremist content has no effect on encoding, storage, or retrieval, it may still have an affect transfer effect on advertising before or after the extremist content (Bergkvist and Taylor, 2016; Wang and Lang, 2012). Viewers may transfer negative feelings about extremist content to the content's pre-roll advertisement and the advertised brand. Alternatively, using a cognitive process, they may have a negative opinion of any brand that supports extremist content with pre-roll advertising. The Nature (2017) study tested this brand-association effect of extremist content.

### 2.2 Video quality

A final variable tested was video quality (Winkler and Faller, 2006). As professionals rarely shoot extremist videos, low production quality, rather than extremism, could be responsible for negative reactions to this content. More widely, if programmatic buying uses classification codes such as V for violence or S for sex, these classifications may group together amateur and professional video. Professional video may be desirable and positive for advertising (as on network television), but amateur video may be undesirable, even for neutral content. For these reasons, this study tested professional (high budget) and amateur (low budget) versions of each content type to evaluate video quality as a programmatic buying parameter.

## 3 Design

A laboratory experiment tested the effects of different types of short video content (less than 5 minutes in duration, like most YouTube videos) on pre-roll advertising. The experiment's participants watched eight short videos, two each (a high- and a low-budget version) of four content types (neutral, violent, sexual, extremist). Before each short video, participants watched a pre-roll ad. The pre-roll ads in this experiment could not be skipped, but to reproduce the typical viewing time for a YouTube pre-roll ad, we used short seven-second ads. A seven-second ad is just longer than the six-second duration of most YouTube pre-roll ads, which are skipped as soon as ad-skipping becomes possible, after five seconds (Campbell et al., 2017). Before each pre-roll ad, participants watched 30-seconds of relaxation video to relax their emotional response after the previous content. After watching all eight short videos, participants completed a questionnaire measuring responses to each video context and then to each pre-roll ad.

This study used a powerful repeated measures design that compares participant content differences rather than group differences (Potter and Bolls, 2012). This design controls for individual difference effects such as age and gender (which varied widely in the non-student sample). As within-participant designs are vulnerable to order effects, randomizing the video presentation-order and the pre-roll ad assignments to the eight videos controlled for order effects. Two sets of eight short videos (both sets had a high and low budget version of four content types, G, V, S, and E) provided replication and generalization, as otherwise significant effects might have been due to the specific experimental stimuli (Potter and Bolls, 2012). Randomly allocated to one of these two video-content groups, one participant group saw one set of eight videos; the other group saw the other set. Both groups saw the same eight pre-roll ads.

## 4 Methods

### 4.1 Sample

The data were collected by MediaScience, a commercial audience laboratory, in accordance with procedures approved by the Murdoch University Human Research Ethics Committee (HREC 2011/157). Informed consent was obtained from all participants, and only the measures an individual had consented to were collected. The 201 participants, representative of the US population, had all consented to watching MA15+ videos but one participant left the session early, leaving a final sample of 200 (50% female, 50% male, aged 19 to 79). All participants earned a $30 American Express gift card for their travel and time. To collect data efficiently, and to generalize the results, the lab sessions ran in identically equipped labs in two centrally located US cities, in Chicago, Illinois and Austin, Texas. There were no demographic differences (gender, age, occupation, income, ethnicity, panel experience), nor brand nor category usage differences for the advertised product categories, between the two video-content groups (measures of these variables were collected at the end of the posttest questionnaire). Finally, there was no program liking difference averaging across the eight different videos that each group saw.

### 4.2 Stimuli

Each participant saw eight videos, a high- and a low-budget example of four different content types: (1) Neutral (G-rated [General audience]), (2) Violence (V, MA15+ [Mature Adults aged 15 years or more]), (3) Sex (S, MA15+), and (4) Extremist (E). The high-budget examples were professional clips from broadcast networks while the low-budget examples were amateur footage. For example, the high-budget neutral videos were scenes from popular US sitcoms, while the low-budget neutral videos were of cute babies or animals. The extremist content related to ISIS (or ISIL). The high-budget extremist content came from CNN or Al Jazeera, while the low-budget content came from a research repository of ISIS videos removed from YouTube. To avoid confounding extremist content with violent content, none of the extremist content was graphically violent (e.g., no beheadings were shown). In Bushman's (2005) study, the violence and sex content came from broadcast network television and rated PG13. This study tested more extreme content from cable networks like HBO and rated MA15+. This extreme content should have increased the chances of finding forward and backward interference effects of online video content.

The eight pre-roll advertisements, the same across both video-content groups, had been in prior advertising effect studies of short commercials (not published yet). Results from these studies were used to select the eight ads for testing interactions between program and advertising content (Kamins et al., 1991). Half of these ads were emotionally neutral, potentially providing a “blank slate” on which the video content could transfer emotional effects (Poncin and Derbaix, 2009). The other half were intensely emotional (two positive and two negative), potentially causing interaction effects with the program content effects. The previous studies measured emotional intensity (arousal) by skin conductance and positive, neutral or negative emotional valence by facial expression (smiling). There were four levels of arousal/valence, each represented by two replications, so that our results would not be confined to four unique ads (Potter and Bolls, 2012). The eight presentation-order variations (Table 1) randomized which pre-roll ad preceded each video. After each video participants watched a 30-second relaxation video to return their arousal to baseline, before seeing the next pre-roll ad.

**Table 1:** Presentation order variations.

Variation	1	2	3	4	5	6	7	8
Video 1	LN1-GL	LN2-GH	LN3-SL	LN4-SH	HN1-VL	HN2-VH	HP1-EL	HP2-EH
Video 2	HN1-GH	HP2-GL	LN4-EL	LN3-VH	LN1-EH	HP1-SH	HN2-SL	LN2-VL
Video 3	LN2-SL	LN1-EL	HP1-GL	HN2-VL	HP2-SH	LN4-EH	LN3-GH	HN1-VH
Video 4	LN3-SH	HP1-VH	LN1-VL	HN1-GL	LN4-SL	HP2-GH	LN2-EH	HN2-EL
Video 5	HP1-VL	LN3-EH	LN2-SH	HP2-SL	HN2-GL	HN1-EL	LN1-VH	LN4-GH
Video 6	LN4-VH	HN2-SH	HN1-EH	LN1-GH	LN3-EL	LN2-GL	HP2-VL	HP1-SL
Video 7	HP2-EL	HN1-SL	HN2-GH	HP1-EH	LN2-VH	LN3-VL	LN4-GL	LN1-SH
Video 8	HN2-EH	LN4-VL	HP2-VH	LN2-EL	HP1-GH	LN1-SL	HN1-SH	LN3-GL

*Notes:* How to read this table: Participants randomly assigned to Variation 1 first saw a low arousal/neutral valence pre-roll ad (LN1) followed by a short video showing low-budget neutral (G-rated) content (GL). The second ad/video combination they saw was a high arousal/negative valence ad (HN1) followed by a high-budget neutral (G-rated) video (GH), etc. Ad Content Types: LN1 = Low Arousal/Neutral Valence Level Ad #1Types: LN2 = Low Arousal/Neutral Valence Level Ad #2Types: LN3 = Low Arousal/Neutral Valence Level Ad #3Types: LN4 = Low Arousal/Neutral Valence Level Ad #4Types: HN1 = High Arousal/Negative Valence Level Ad #1Types: HN2 = High Arousal/Negative Valence Level Ad #2Types: HP1 = High Arousal/Positive Valence Level Ad #1Types: HP2 = High Arousal/Positive Valence Level Ad #2Types: Video Content Types:Types: GL = Neutral, Low BudgetTypes: GH = Neutral (G-Rated), High BudgetTypes: VL = Violence, Low BudgetTypes: VH = Violence (V-Rated, MA15+), High BudgetTypes: SL = Sex, Low BudgetTypes: SH = Sex (S-Rated, MA15+), High BudgetTypes: EL = Extremist, Low BudgetTypes: EH = Extremist, High Budget

### 4.3 Measures

The questionnaire that measured ad effectiveness began, in line with the cover story for the study, by measuring program liking for each short video by the average of three Likert items (Coulter, 1998). Bushman's (2005) single-item manipulation check measures confirmed whether each video contained violent or sexual content. A new item developed for this study measured whether the video contained extremist content (“This video was about ISIS/ISIL”).

#### 4.3.1 Dependent variables

The advertising effectiveness measures were collected after the video content measures. The first effectiveness measure was unaided (free) brand recall, collected before mentioning any brand names in the questionnaire. Participants were asked to: “Please list the names of the brands and products (e.g., Dove body wash) that you remember seeing or hearing ads for in today's session.” Correct brand recall (slight misspellings allowed) was coded as 1, otherwise 0. Next, aided (cued) recall was collected by showing the product categories associated with the eight test brands (bottled water, toilet cleaners, insurance, snack chips, pet snacks, family-style restaurants, travel accommodation, and refrigerators), and asking: “Do you remember the brands that were advertised for each category?” Again, correct brand recall was coded 1, otherwise 0. Brand recognition was collected by showing participants a randomized list of the eight brand names and their closest competitors (i.e., 16 total brands, so the guess rate was 50%) and asking participants: “Can you identify brands that were advertised during your session from the list below? Some are brands you actually saw and some are decoys.” Correct brand recognition was coded 1, otherwise 0. Ad liking (Bergkvist and Rossiter, 2007), brand attitude (Bergkvist and Rossiter, 2007), and purchase intention (Juster, 1966; Wright et al., 2002) were validated single item measures (see S1.SupplementaryTable.docx and S2.SurveyData.csv).

#### 4.3.2 Biometric measures

Biometric measures of skin conductance and facial expression (smiling) were collected during the viewing experience (see S3.Biometrics.csv). Skin conductance was via two electrodes attached to the index and middle finger of each consenting participant. Sweating, due to an increase in emotional arousal, increases the flow of electricity (i.e., skin conductance) between these two electrodes (Potter and Bolls, 2012). Since analysis of variance (ANOVA) and other parametric tests assume the dependent variable is normally distributed (Kirk, 1995), the initially skewed skin conductance data were log transformed so that they were normally distributed.

A high definition camera recorded each consenting participant's facial expression for subsequent computer analysis (FACET, from www.iMotions.com). The software estimated the participant's smiling probability on a second-by-second basis. Ideally, the same software could have been used to measure negative emotional response (e.g., frowning) during the negative valence pre-roll ads, but currently computers have limited ability to detect facial expressions other than smiling. Instead, negative valence was implied by the absence of smiling.

## 5 Results

### 5.1 Manipulation checks

Table 2 lists the means for the four video content types. Video content effects were examined with a 4 × 2 × 2 (Content [neutral, violence, sex, extremist] × Budget [low, high] × Group [1, 2]) analysis of variance (ANOVA). The violent content videos were rated significantly more violent than the other content, the sex content videos rated significantly more sexually arousing and finally, the extremist content rated significantly more ISIS related. Group and budget interaction effects qualified these main effects (results available from the authors, because group and budget had no significant effects on the dependent variable measures of advertising effectiveness). For example, one group saw videos more violent than the videos the other group saw (*M*_1_ = 3.92 vs. *M*_2_ = 5.04, *F*(1, 198) = 30.74, *p* < .001, η_p_^2^ = .13). The low budget videos were rated more violent than the high budget videos (*M*_low_ = 4.17 vs. *M*_high_ = 4.79, *F*(1, 198) = 49.70, *p* < .001, partial η^2^ = .20). High budget sex content was rated sexier than low-budget content. One group rated the violent videos more ISIS related than the other group did. The main analyses controlled for these main and interaction effects of content. The estimated marginal means in Table 2 control for these program content effects.

**Table 2:** Means for video content types.

Variable	Neutral (G)	Violent (V)	Sex (S)	Extremist (E)	Test of content effect
Violence (1–10)	3.06^a^ (.15)	8.14^b^ (.12)	1.78^c^ (.12)	4.94^d^ (.19)	*F*(3, 196) = 512.26, *p* < .001, η_p_^2^ = .89
Sex (1–10)	2.46^a^ (.14)	1.32^b^ (.09)	5.44^c^ (.18)	1.20^b^ (.08)	*F*(3, 196) = 181.78, *p* < .001, η_p_^2^ = .74
Extremist (1–10)	3.42^a^ (.19)	2.25^b^ (.14)	1.76^c^ (.14)	8.36^d^ (.16)	*F*(3, 196) = 367.14, *p* < .001, η_p_^2^ = .85
Arousal (μSiemens) (during video content)	−.02^a^ (.04)	.06^b^ (.03)	.06^b^ (.04)	−.03^a^ (.04)	*F*(3, 194) = 3.48, *p* = .02, η_p_^2^ = .05
Arousal (μSiemens) (during pre-roll ad)	.04^a^ (.02)	.04^a^ (.02)	.08^a^ (.02)	.05^a^ (.03)	*F*(3, 194) = 1.96, *p* = .12, η_p_^2^ = .03
Valence (smiling prob.) (during video content)	83.1^a^ (.03)	43.3^b^ (.02)	56.5^c^ (.03)	30.1^d^ (.02)	*F*(3, 195) = 35.95, *p* < .001, η_p_^2^ = .36
Valence (smiling prob.) (during pre-roll ad)	58.8^a^ (.02)	53.8^a^ (.02)	54.8^a^ (.02)	52.8^b^ (.02)	*F*(3, 195) = 1.61, *p* = .19, η_p_^2^ = .02
Free brand recall (%)	25.0^a^ (.02)	23.5^a^ (.02)	22.5^a^ (.02)	21.3^a^ (.02)	*F*(3, 196) = .58, *p* = .63, η_p_^2^ = .01
Cued brand recall (%)	50.0^a^ (.03)	45.8^a^ (.03)	45.5^a^ (.03)	49.0^a^ (.03)	*F*(3, 196) = .94, *p* = .42, η_p_^2^ = .01
Brand recognition (%)	70.0^a^ (.02)	64.3^a^ (.03)	66.8^a^ (.02)	63.0^a^ (.03)	*F*(3, 196) = 1.88, *p* = .14, η_p_^2^ = .03
Ad liking (1–6)	4.50^a^ (.07)	4.43^a^ (.07)	4.39^a^ (.06)	4.52^a^ (.06)	*F*(3, 196) = 1.70, *p* = .17, η_p_^2^ = .02
Brand attitude (1–6)	4.44^a^ (.08)	4.39^a^ (.08)	4.38^a^ (.08)	4.53^a^ (.08)	*F*(3, 196) = 1.02, *p* = .38, η_p_^2^ = .02
Purchase intention (%)	37.7^a^ (.02)	33.5^a^ (.02)	36.1^a^ (.02)	37.9^a^ (.02)	*F*(3, 196) = 2.34, *p* = .07, η_p_^2^ = .04

Notes: *N* = 200. Standard errors in parentheses. Means in the same row with the same superscript letter are not significantly different from each other (*p* < .05).

Differences in video quality ratings and program liking checked the high versus low budget video quality manipulation (again, these results are available from the authors). Relative to low budget videos, high budget videos rated higher in video quality (*M*_high_ = 74.7% vs. *M*_low_ = 61.3%, *F*(1, 198) = 149.96, *p* < .001, η_p_^2^ = .43) and liking (*M*_high_ = 3.56 vs. *M*_low_ = 2.57, *F*(1, 198) = 230.92, *p* < .001, η_p_^2^ = .54). Budget also made a significant difference to content familiarity (whether it had been seen before). Again, content and group interactions qualified these main effects. For example, the extremist content in one group was rated as much lower in video quality. The gap between liking low- and high-budget content was wider for sexual content videos. Again, the main analyses control for these effects.

The differences between the pre-roll ads were checked by skin conductance (measuring arousal) and smiling (measuring valence). Ad content effects were examined with a 4 × 2 × 2 × 8 (Arousal/Valence [low/neutral 1, low/neutral 2, high/negative, high/positive] × Replication [1, 2] × Group [1, 2] × Variation [1–8, i.e., the effects of video content and budget]) ANOVA. There were significant differences in arousal and valence between the four different types of ad content (Table 3). Two of the low-arousal/neutral-valence ads (LN1 and LN2) were significantly less arousing than the high-arousal/positive-valence ads. The high-arousal/positive-valence ads had a significantly higher probability of smiling than the other three levels of arousal/valence.

**Table 3:** Means for ad content types.

Variable	Low arousal/neutral valence (1)		Low arousal/neutral valence (2)		High arousal/negative valence		High arousal/positive valence		Test of content effect
Arousal/valence	1		2		3		4		
Replication	1	2	1	2	1	2	1	2	
Ad	LN1	LN2	LN3	LN4	HN1	HN2	HP1	HP2	
Arousal (μSiemens) (log-transformed)	1.18^a^ (.07)		1.24^b^ (.06)		1.21^a,b^ (.07)		1.22^b^ (.07)		*F*(3, 179) = 4.41, *p* = .005, η_p_^2^ = .07
Valence (smiling prob.)	38.5^a^ (.04)		40.2^a^ (.04)		36.2^a^ (.04)		63.4^b^ (.05)		*F*(3, 170) = 23.00, *p* < .001, η_p_^2^ = .29
Free brand recall (%)	31.7^a^ (.02)		12.8^b^ (.02)		25.0^c^ (.02)		23.5^c^ (.02)		*F*(3, 182) = 22.76, *p* < .001, η_p_^2^ = .27
Cued brand recall (%)	55.8^a^ (.03)		33.1^b^ (.02)		54.1^a^ (.03)		47.4^c^ (.03)		*F*(3, 182) = 26.15, *p* < .001, η_p_^2^ = .30
Brand recognition (%)	81.4^a^ (.02)		52.0^b^ (.02)		77.5^a^ (.02)		53.0^b^ (.02)		*F*(3, 182) = 73.20, *p* < .001, η_p_^2^ = .55
Ad liking (1–6)	4.23^a^ (.07)		4.60^b^ (.06)		4.39^c^ (.06)		4.61^b^ (.07)		*F*(3, 182) = 13.12, *p* < .001, η_p_^2^ = .18
Brand attitude (1–6)	4.20^a^ (.09)		4.69^b^ (.07)		4.53^c^ (.08)		4.31^a^ (.08)		*F*(3, 182) = 14.50, *p* < .001, η_p_^2^ = .19
Purchase intention (%)	41.1^a^ (.02)		39.6^a^ (.02)		33.5^b^ (.02)		30.8^b^ (.02)		*F*(3, 182) = 13.72, *p* < .001, η_p_^2^ = .18

Notes: *N* = 200. Standard errors in parentheses. Means in the same row with the same superscript letter are not significantly different from each other (*p* < .05).

### 5.2 Main results

Video content showed no significant effects on any advertising effectiveness measure: free brand recall, cued brand recall, brand recognition, ad liking, brand attitude, or purchase intention (Table 2). The ANOVA results in Table 4 show no significant main effects of presentation-order variation (Table 1). A sequential mediation (model 6) test using PROCESS v.3 (Hayes, 2018) showed no mediating effect of variation → ad liking → brand attitude → purchase intention, mainly because there was no overall effect of the independent variable (variation) on the dependent variable (purchase intention) (*F*(7, 192) < 1, *p* = .66). For each variation, the 95% confidence interval (CI) for the partially standardized indirect sequential mediation effect included zero (variations 2 to 8 [1 was the default]: −.12, .05; −.12, .08; −.14, 11; −.08, .11; −.05, .15; −.10, .11; −.04, .16). The manipulation checks confirmed that high versus low budget (another characteristic of video content) made a significant difference to video quality, program liking, and program familiarity ratings. But budget made no significant difference to measures of ad effectiveness (the largest effect of budget was on free brand recall and it was not significant, *F*(1, 198) = 3.33, *p* = .07, partial η^2^ = .02).

**Table 4:** ANOVA results.

Variable	Free brand recall	Cued brand recall	Brand recognition	Ad liking	Brand attitude	Purchase intention
*Between groups*						
Intercept	.58***	.76***	.90***	.98***	.97***	.79***
Group (G)	<.001	.002	.001	.02	.002	.01
Variation (V)	.01	.02	.03	.02	.05	.03
G × V	.07	.02	.01	.02	.02	.02
*Within-participants*						
Arousal/valence (AV)	.09***	.12***	.28***	.07***	.06***	.07***
AV × G	.003	.01	.004	.01	.01	.003
AV × V	.08***	.06	.04	.03	.04	.01
AV × G × V	.05	.04	.04	.03	.03	.03
Replication (R)	.17***	.11***	.02	.03*	.14***	.17***
R × G	.001	.003	.004	<.001	<.001	.003
R × V	.12**	.03	.02	.03	.02	.02
R × G × V	.05	.04	.04	.03	.01	.05
AV × R	.05***	.18***	.34***	.08***	.23***	.22***
AV × R × G	<.001	.01	.01	.004	.002	.004
AV × R × V	.09***	.02	.04	.04	.08**	.06
AV × R × G × V	.03	.04	.04	.02	.06	.05

*Notes:***Significant effects in bold.** Numbers shown are effect sizes (partial η^2^), small = .01, medium = .06, large = .14 (Cohen, 1988). *Group* tests the difference between the two sets of eight short videos. *Variation* tests the effects of presentation order variation (Table 1). *Arousal/Valence* had 4 levels, low-arousal/neutral valence (levels 1 and 2), high-arousal/negative valence (level 3), high-arousal/positive valence (level 4). *Replication* and AV×R test unique brand effects of the two ads at each AV level.

*p < .05, ** *p* < .01, *** *p* < .001.

One explanation for finding no effects of video content on pre-roll advertising effectiveness is that this study was underpowered and unable to detect any differences in advertising effectiveness. But there were significant effects of ad content (arousal/valence) on all six measures of advertising effectiveness (Tables 3 and 4). In addition, a simple mediation (model 4) test using PROCESS showed, as would be expected, a significant mediating effect of brand attitude between ad liking and purchase intention (95% CI of the standardized indirect effect: .06, .29).

**Fig. 1:**
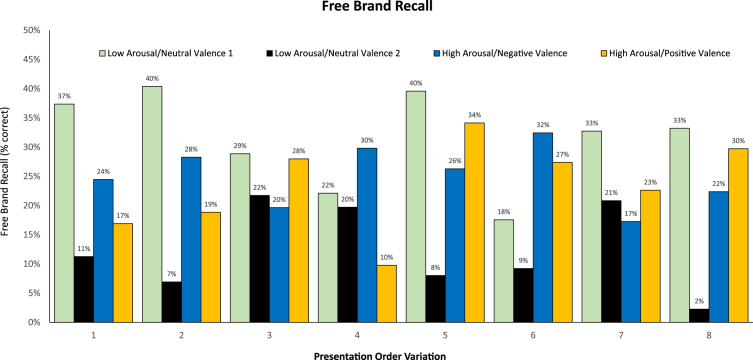
Interaction between arousal/valence and presentation-order variation.

Another potential explanation for finding no main effect of video content on pre-roll advertising effectiveness is that these effects are confined to certain types of video content, when combined with certain types of pre-roll ad content. This study was designed to test this possibility, by examining the two-way interactions between presentation-order variation and the arousal/valence of the pre-roll ad. There was one significant two-way interaction effect, on free brand recall (Fig. 1). However, there was no pattern to these interaction results. Overall, the second set of low-arousal/neutral-valence ads (LN3 and LN4, black columns in Fig. 1) had the lowest level of free brand recall, and this level was significantly lower compared with the other three levels of arousal/valence (Table 3).

But for three presentation order variations (3, 4, and 7), the pattern of results differed. In these three variations, the second set of low arousal/neutral valence ads did not have the lowest level of free brand recall, and there were no significant differences between levels of arousal/valence (Fig. 1). Looking at the types of video content associated with these ads in these three presentation orders (variations 3,4, and 7 in Table 1), there was no systematic relationship between program content and this different pattern of results. In these three presentation order variations, these two low-arousal/neutral valence ads (LN3 and LN4) associated with all four types of content (G, V, S, and E) and both levels of budget (L and H). The explanation for their higher level of recall in these presentation order conditions compared with the others is more likely a recency effect, as in two of these three variations (variations 3 and 4) these ads were seen before the first and second videos.

**Fig. 2:**
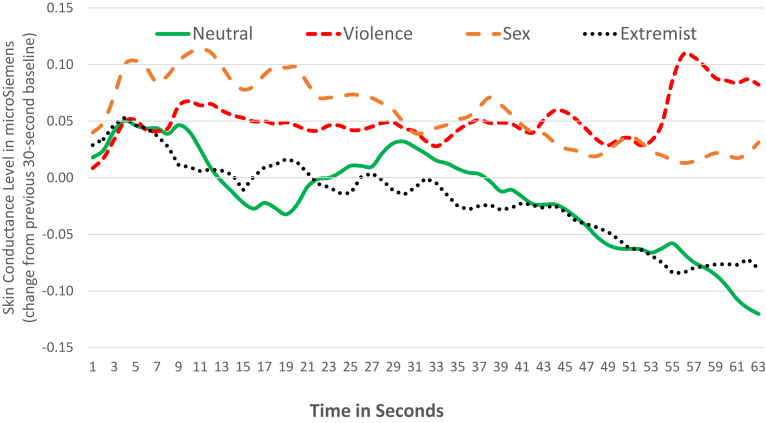
Emotional arousal over time.

In mid-roll advertising research, forward spillover effects of emotional arousal from the program content to the advertising in the breaks explained the content effects on the advertising. The present study showed no corresponding backwards spillover effects of program content on pre-roll advertising. Fig. 2 shows differences among the four content types in their emotional arousal, measured by skin conductance, during the first 63 seconds of ad and program content (i.e., the first seven seconds of each line is the pre-roll ad). As shown in Fig. 2, the general tendency is for skin conductance to decline over time as participants get more tired or relaxed.

To compare differences between types of program content, rather than differences caused by this trend over time, the effect of this decline in skin conductance was removed by converting the raw skin conductance scores into change scores (Potter and Bolls, 2012). These change scores measured the difference between each second of content and the mean skin conductance during the last five seconds of the most recent baseline period. There was a 30-second baseline period after each program video and before each pre-roll ad. For example, if the mean skin conductance during the last five seconds of the previous baseline was 4.0 microSiemens and skin conductance during the first second of the pre-roll after this baseline was 4.2 microSiemens, the change score for that second would be 4.2 minus 4.0 = 0.2 microSiemens.

The differences between the four content types in Fig. 2 were tested by simple ANOVA tests. To avoid multiple testing and reporting chance results, nine ANOVA tests rather than 63 were carried out, using averages for the nine seven-second periods in Fig. 2. The significance level was set at *p* < .001. These tests revealed that sex content was marginally higher (*p* < .05) in arousal compared with the other three types of content during the second period (8–14 s) and significantly higher (*p* < .001) than neutral content during the third period (15–21 s) and extremist content during the fourth period (22–28 s). There were no significant differences during the next three periods (29–49 s). In the last two periods (50–63 s), violence was more arousing than neutral and extremist content.

**Fig. 3:**
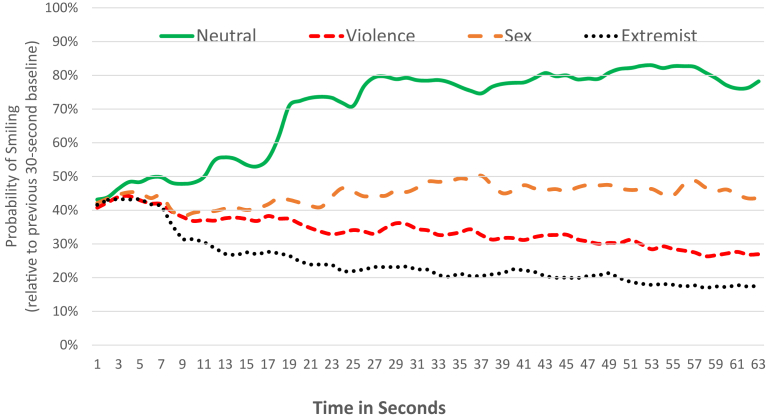
Smiling probability over time.

Smiling probability results showed a different pattern (Fig. 3). Smiling was significantly higher during the neutral content (i.e., situation comedies and cute babies) than during the other video content from the second period (8–14 s) onwards. Extremist content had significantly less smiling, compared to other content from the third period (15–21 s) onwards. From period four (22–28 s) onwards, there were significant differences between all four types of content. But there were no backwards spillover effects of smiling from the video content to the pre-roll advertising.

## 6 Discussion

This laboratory study tested whether short video content, such as that seen on YouTube, can have effects on pre-roll advertising. The study tested extremist content effects, because brands have pulled their advertising from YouTube fearing ‘brand safety’ harm if their ads appear before extremist content. The study also tested violent and sexual content's potential effects, which have been significant on mid-roll advertising (Bushman, 2005).

In this study, program content had no effect on pre-roll ad effectiveness. There was no brand safety effect on the brand's reputation, measured by brand attitude. These results suggest that the content seen after a pre-roll ad does not interfere with processing that ad, even controversial—violent, sexually arousing, or extremist—content following the ad.

### 6.1 Theoretical contribution

The study's main theoretical contribution is suggesting that video content has no effect on pre-roll ad effectiveness. This pre-roll study, like the Nature (2017) pre-roll study, found no video context effects on pre-roll ads seen before the video content. Extremist content, violent content, sexual content, and high versus low budget content, made no significant difference. Like the Nature (2017) study, brand attitude tested for low- and high-involvement effects of extremist content on a brand's reputation.

These results contrast with mid-roll advertising research showing that programs rated S for sex or V for violence interfere with processing of 30-second commercials in mid-roll breaks (Bushman and Bonacci, 2002; Bushman, 2005). Together, these results suggest that program content can have forward spillover effects on ads that follow the content, but no interference effects on ads seen before the content.

High arousal spilling over from the program onto ads in the break can explain sex or violent content's effects on mid-roll ads. Rather than processing the incoming advertising content, program-induced arousal causes and reflects distracted thinking about the violent or sexual content seen prior to the ad break. Violence and sex distract automatically because they have high biological relevance (Lang, 2017; Lang et al., 2005). But this distraction effect seems irrelevant to pre-roll ads. As pre-roll ads appear before the video content, there can be no *forward* spillover effects of program content onto the ads.

The study measured arousal using a biometric, skin conductance, and found no arousal differences caused by program content during the pre-roll ads. Sexual and violent content was significantly more arousing than neutral content. The study's chosen extremist videos featured no violence, to isolate the effect of violence. Potentially, negative reactions to this extremist content may have elevated emotional arousal measured by skin conductance. But in this study, extremist videos were only as arousing as neutral videos.

Research has suggested that novel, complex, or biologically relevant stimuli can distract cognitive processing resources away from the memory processes of encoding, storage, and retrieval (Lang, 2017; Lang et al., 2005, 2013). To keep up with constantly changing video content, most cognitive resources are deployed to encoding rather than storage and retrieval, which means that encoded memories of content previously seen are potentially not stored effectively and therefore not retrievable using cued or free recall tasks. When the current content is violent and therefore highly distracting, eliciting attention responses and thinking about this current content, previously encoded content is potentially even less likely to be stored effectively and recalled later (Lang et al., 1996). In the present study's results, all three memory processes were similarly unaffected by video content seen after a pre-roll ad. This suggests that strongly violent and sexual content, and extremist content, does not distract cognitive resources away from adequate storage and retrieval of pre-roll advertising. Pre-roll ads had equal brand recognition (encoding), cued brand recall (storage), and free brand recall (retrieval) whether seen before neutral G-rated content, or violent (V), sexual (S), or extremist (E) content.

Ad liking, sensitive to affect-valence transfer effects (Poncin and Derbaix, 2009), has downstream effects on brand attitude and purchase intention (Pham et al., 2013). This study, however, found no significant differences on these variables among ads seen before any of the four types of video content.

Using content that was too mild, compared to content seen on YouTube, cannot explain these null results. The content was more extreme than the content in Bushman's (2005) mid-roll study, which had significant effects on advertising effectiveness. On the same 10-point scales, his sex content rated 3.6 (vs. this study's 5.4), and his violence content rated 5.8 (vs. 8.1 in this study).

### 6.2 Limitations and suggestions for future research

More research should confirm and explain this study's results showing insignificant effects of any content—violent, extremist, sexual, or neutral—on measures of pre-roll ad effectiveness. The primary limitation is that this study did not compare pre-roll advertising with mid-roll advertising to replicate previously published findings. It is possible that the lab experiment procedures used in the current experiment would not have detected significant mid-roll advertising effects either. In the present study's laboratory experiment, attention to the content and the advertising was higher than outside the lab. In prior mid-roll studies, the viewing environment was more relaxed (Bushman and Bonacci, 2002; Bushman, 2005). Participants in those mid-roll studies watched the content in small groups, with snacks available at the back of the room. Potentially, that distracting environment explains the significant results found in those studies, as viewers could do something else other than look at the advertisements during ad breaks.

Future research could test the effects of video content on pre-roll advertising when viewed in groups on a large screen. However, the results of that study would only apply to YouTube viewing on a connected TV. The majority of YouTube viewing is likely to be individual viewing, on a small screen, like the conditions in this lab study.

Finally, this study used biometrics and single-item measures of, for example, ad liking and brand attitude, to assess effects on emotional responses to different types of program content. It is likely, however, that the effects of extremist content on pre-roll advertising have nothing to do with how people feel, but how they think. Future studies should use qualitative research, such as talk aloud protocols, interviews, and ethnography to understand the thought processes potentially reponsible for program content harming the reputation of a brand in a pre-roll ad (Karimi et al., 2018).

## 7 Conclusions

This study suggests brand safety is not an issue for pre-roll ad effectiveness. Video content had no interference effects on ads seen before program content. A brand's reputation might suffer negative effects from pre-roll advertising in other ways, however. Journalists could report that the brand has (accidentally) supported extremist groups with pre-roll ad income. This would be a brand scandal effect via the media rather than an effect on the (few) consumers who saw the ad before the extremist content.

The chances seem unlikely that a pre-roll ad will run before an extremist video. Few extremist video channels would pass the criteria for audience size necessary to gain advertising. YouTube channels must have over 1,000 subscribers and 4,000 watch hours over the previous year to apply for monetization (Murphy et al., 2018). The effect of advertising on these channels should mostly be positive and might be the best way of reaching some hard-to-reach consumers. Nevertheless, concerned advertisers could use an E (extremist) content parameter when using programmatic buying. Artificial intelligence might allow the rapid and accurate identification of all extremist content.

## Declarations

### Author contribution statement

Steven Bellman: Conceived and designed the experiments; Analyzed and interpreted the data; Wrote the paper.

Ziad H. S. Abdelmoety, Jamie Murphy: Analyzed and interpreted the data; Wrote the paper.

Shruthi Arismendez: Performed the experiments; Contributed reagents, materials, analysis tools or data.

Duane Varan: Conceived and designed the experiments; Contributed reagents, materials, analysis tools or data.

### Funding statement

This work was supported by the sponsors of the Beyond: 30 project, as Study 71 “Does program context affect pre-roll ads?”.

### Competing interest statement

SB, SA, and DV's research is sponsored by a consortium of companies that includes Google (owned by Alphabet like YouTube) but also Google's competitors, such as television networks and Facebook. The authors therefore believe that their research is independent, not influenced by any single sponsor or industry group.

### Additional information

No additional information is available for this paper.

## Appendix A Supplementary data

S1.Supplementary Table S1.Supplementary Table

S2.Survey Data S2.Survey Data

S3.Biometrics Data S3.Biometrics Data

## References

[bib1] Bergkvist L., Rossiter J.R. (2007). The predictive validity of multiple-item versus single-item measures of the same constructs. J. Market. Res..

[bib2] Bergkvist L., Taylor C.R. (2016). Leveraged marketing communications: a framework for explaining the effects of secondary brand associations. AMS Rev..

[bib3] Bushman B.J. (2005). Violence and sex in television programs do not sell products in advertisements. Psychol. Sci..

[bib4] Bushman B.J., Bonacci A.M. (2002). Violence and sex impair memory for television ads. J. Appl. Psychol..

[bib5] Cacioppo J.T., Berntson G.G., Norris C.J., Gollan J.K., Van Lange P.A.M., Kruglanski A.W., Higgins E.T. (2012). The evaluative space model.

[bib6] Campbell C., Thompson F.M., Grimm P.E., Robson K. (2017). Understanding why consumers don't skip pre-roll video ads. J. Advert..

[bib7] Christianson S.-Å. (2017). Emotional stress and eyewitness memory: a critical review. Psychol. Bull..

[bib8] Cohen J. (1988). Statistical Power Analysis for the Behavioral Sciences.

[bib9] Coulter K.S. (1998). The effects of affective responses to media context on advertising evaluations. J. Advert..

[bib10] Fulgoni G.M., Lipsman A. (2017). Measuring television in the programmatic age: why television measurement methods are shifting towards digital. J. Advert. Res..

[bib11] Hayes A.F. (2018). Introduction to Mediation, Moderation, and Conditional Process Analysis: a Regression-Based Approach.

[bib12] Hickman A. (2017). YouTube Revenues Estimated to Take 7.5% Hit in 2017.

[bib13] Juster F.T. (1966). Consumer buying intentions and purchase probability: an experiment in survey design. J. Am. Stat. Assoc..

[bib14] Kahneman D., Tversky A. (1979). Prospect theory: an analysis of decision under risk. Econometrica.

[bib15] Kamins M.A., Marks L.J., Skinner D. (1991). Television commercial evaluation in the context of program-induced mood: congruency versus consistency effects. J. Advert..

[bib16] Karimi S., Holland C.P., Papamichail K.N. (2018). The impact of consumer archetypes on online purchase decision-making processes and outcomes: a behavioural process perspective. J. Bus. Res..

[bib17] Kirk R.E. (1995). Experimental Design: Procedures for the Behavioral Sciences.

[bib18] Lang A., Rössler P., Hoffner C.A., van Zoonen L. (2017). Limited capacity model of motivated mediated message processing (LC4MP). The International Encyclopedia of Media Effects.

[bib19] Lang A., Newhagen J., Reeves B. (1996). Negative video as structure: emotion, attention, capacity, and memory. J. Broadcast. Electron. Media.

[bib20] Lang A., Chung Y., Lee S., Zhao X. (2005). “It’s the product: do risky products compel attention and elicit arousal in media users?. Health Commun..

[bib21] Lang A., Sanders-Jackson A., Wang Z., Rubenking B. (2013). Motivated message processing: how motivational activation influences resource allocation, encoding, and storage of TV messages. Motiv. Emot..

[bib22] Lee S., Lang A. (2009). Discrete emotion and motivation: relative activation in the appetitive and aversive motivational systems as a function of anger, sadness, fear, and joy during televised information campaigns. Media Psychol..

[bib23] Lull R.B., Gibson B., Carlos Cruz C., Bushman B.J. (2018). Killing characters in video games kills memory for in-game ads. Psychol. Popular Media Cult..

[bib24] Maheshwari S. (2017). On YouTube Kids, Startling Videos Slip Past Filters.

[bib25] Mattes J., Cantor J. (1982). Enhancing responses to television advertisements via the transfer of residual arousal from prior programming. J. Broadcast..

[bib26] Murphy P.P., Yurieff K., Mezzofiore G. (2018). Exclusive: YouTube Ran Ads from Hundreds of Brands on Extremist Channels. http://money.cnn.com/2018/04/19/technology/youtube-ads-extreme-content-investigation/.

[bib27] Nature Pty Ltd (2017). YouTube Boycott: Is it All a Storm in a Teacup?. https://www.natureresearch.com.au/2017/04/youtube-boycott-is-it-all-a-storm-in-a-teacup/.

[bib28] Öhman A., Flykt A., Esteves F. (2001). Emotion drives attention: detecting the snake in the grass. J. Exp. Psychol. Gen..

[bib29] Pham M.T., Geuens M., De Pelsmacker P. (2013). The influence of ad-evoked feelings on brand evaluations: empirical generalizations from consumer responses to more than 1000 TV commercials. Int. J. Res. Market..

[bib30] Poncin I., Derbaix C. (2009). Commercials as context for other commercials. J. Advert..

[bib31] Potter R.F., Bolls P.D. (2012). Psychophysiological Measurement and Meaning: Cognitive and Emotional Processing of media.

[bib32] Potter R.F., LaTour M.S., Braun-LaTour K.A., Reichert T. (2006). The impact of program context on motivational system activation and subsequent effects on processing a fear appeal. J. Advert..

[bib33] Wakabayashi D., Maheshwari S. (2017). YouTube Advertiser Exodus Highlights the Perils of Online Ads.

[bib34] Wang Z., Lang A. (2012). Reconceptualizing excitation transfer as motivational activation changes and a test of the television program context effects. Media Psychol..

[bib35] Winkler S., Faller C. (2006). Perceived audiovisual quality of low-bitrate multimedia content. IEEE Trans. Multimed..

[bib37] Wright M., Sharp A., Sharp B. (2002). Market statistics for the Dirichlet model: using the Juster scale to replace panel data. Int. J. Res. Market..

