# Supplementary material for: Brand safety: the effects of controversial video content on pre-roll advertising
Source: Heliyon. 2018 Dec 19;4(12):e01041. doi: 10.1016/j.heliyon.2018.e01041 (PMC6304455; doi:10.1016/j.heliyon.2018.e01041)
Supplement: S1.Supplementary Table [file mmc1.docx]

# Supplementary Table

## Questionnaire Items

*Program Familiarity:*

Have you seen this video content before? No = 0, Yes = 1.

*Program Liking* (3 items, α = .96; 1 = Strongly Disagree to 6 = Strongly Agree)

I’m glad I had a chance to see this video content.

If I knew this content was available, I would look forward to watching it.

I think this is video content I would be interested in watching in the future.

(Coulter, 1998)

*Content Manipulation Checks* (Not At All = 1 to Extremely = 10)

How violent did you think this video was?

How sexually arousing did you think this video was?

This video was about ISIS/ISIL.

(Bushman, 2005, except last item)

*Video Quality*

Your opinion of the program may have been affected by the quality of the video.

Please rate the quality of the video you saw (program plus ads):

The quality of the video was (Bad = 0 to Excellent = 100).

(Winkler and Faller, 2006).

*Free Brand Recall, Cued Brand Recall, and Brand Recognition*

See pages 13 and 14 for descriptions of these measures.

*Ad Liking*

Thinking about this ad for BRANDNAME, which of the following statements best describes your feelings about this ad?

I Disliked It Very Much = 1 to I liked it very much = 6

(Bergkvist and Rossiter, 2007)

*Brand Attitude*

What is your attitude toward BRANDNAME as a brand of CATEGORY?

Bad = 1 to Good = 6.

(Bergkvist and Rossiter, 2007)

*Purchase Intention*

Using the following scale, and taking everything into account, what are the chances that you, personally, will buy something from BRANDNAME in the next 6 months? [3 months if a regularly purchased item]

No chance = 1 (0%) to Certain or practically certain = 11 (99%)

(Juster, 1966; Wright *et al.,* 2002)
